# Industrial Health

**Published:** 1921-05-07

**Authors:** 


### Industrial Health

### WHAT WELFARE WORK IS DOING.

We have received from the editor of the journal published by the Industrial Welfare Society an interesting account of the work already done in firmly establishing the welfare principle in British industry. The Society is now continually receiving requests from firms for definite assistance in the application of the principal to their particular needs, and it is now organising its activities to this end. We gladly accede to the request to give publicity to the purpose and character of this organisation, whose functions, we are well aware, are still misunderstood in some quarters in which it seems to be imagined that the Society exists chiefly to "make things easy" for employers. What, then, is industrial welfare work? It is a movement in which leading representatives of all classes of the community are co-operating for furthering the cause of industrial peace "by helping to secure for all workers the best possible conditions of working; recognition of their needs in connection with all those matters affecting their well-being which are not covered by negotiation between employers' federations and trade unions; and by increasing in a practical way understanding and goodwill all round. While the promotion of efficiency is not the motive force behind the movement, it can scarcely be denied that ultimately the best worker is the contented worker."

**THE PUBLIC WELL-BEING**—(*continued*)

The exact application of welfare principles, it is pointed out, varies with the circumstances of industries and firms, but the movement is concerned with questions of housing, transport, engagement, progress, dismissal, health, working conditions, canteens, sanitary and lavatory arrangements, sickness and convalescence, education, thrift, recreation, and social activities. The introduction of organised schemes of welfare work provides a means, perhaps the best means, of bringing together in a common purpose all the sections of a business undertaking, and produces what has been aptly described as "esprit-de-firm." Public attention was attracted to the possibilities of the movement in the early days of the war by the action of the Ministry of Munitions in setting up a Welfare Department. Just over two years ago the Industrial Welfare Society was formed by a number of well-known industrial leaders for the purpose of continuing upon a voluntary basis the work then started by the State, and during the short period which has since elapsed its work has attracted worldwide attention.

The Society exists for the purpose of serving employers and others, and, along the following broad lines, places its organisation at the disposal of those who may desire to use it:—

By assisting employers to introduce organised schemes of welfare work.

By providing men and women competent to undertake the task of welfare supervision.

By retaining interest and maintaining progress by means of lectures, visits, literature, etc.

By supplying firms with information and by pooling the experiences of others in all parts of the world.

By offering expert advice in all matters relating to schemes of welfare work.

The very wide publicity now being given to the movement is due, we are reminded, almost entirely to the operations of the Society, and, although the results in this direction have been eminently satisfactory, the Society itself has suffered. Greater interest has been secured for the interests of the welfare movement, but the claims of the Society for recognition and support have been overlooked, and the entirely false impression has gained ground in many quarters that its finances are in a thoroughly sound position and in need of no further assistance. But the warning is here conveyed that, if this beneficent work—beneficent not only from the point of view of health, but in promoting peace and goodwill in industry—is to be continued and developed, a much greater measure of support must be forthcoming from those who desire to retain welfare work for industry, "instead of allowing it to become controlled and directed by rigid official methods." The choice, it is frankly declared, must be made, and made soon, for the movement is bound to continue under one direction or the other. Up to the present time the Society has depended for its existence on the goodwill of a few employers, and whilst its activities have been approved by firms in every industry their approval has not been backed so far by financial assistance. We hope the warning and appeal thus conveyed will have the desired effect, for we know of no voluntary public effort in this country more worthy of being set up on an established basis, for there is none with such far-reaching possibilties for improving the lot of the worker by raising his standard of health, improving his working conditions, and humanising the relations between master and man.

